# An adolescent with both Wegener's Granulomatosis and chronic blastomycosis

**DOI:** 10.1186/1546-0096-6-13

**Published:** 2008-08-03

**Authors:** Reem Abdwani, Kristin Houghton, Simon Dobson, Maureen O' Sullivan, Peter Malleson

**Affiliations:** 1 Division of Child health, Sultan Qaboos University Hospital, Muscat, Oman; 2 Division of Pediatric Rheumatology, University of British Columbia, Vancouver, Canada; 3 Division of Infectious Disease, University of British Columbia, Vancouver, Canada; 4 Department of Pathology & Laboratory Medicine, University of British Columbia, Vancouver, Canada

## Abstract

We report a case of Wegener's Granulomatosis (WG) associated with blastomycosis. This appears to be the first case report of WG co-existing with a tissue proven blastomycosis infection. The temporal correlation of the two conditions suggests that blastomycosis infection (and therefore possibly other fungal infections), may trigger the systemic granulomatous vasculitis in a predisposed individual; a provocative supposition warranting further study.

## Introduction

Wegener's Granulomatosis is a systemic small vessel vasculitis of unknown etiology, associated with the presence of anti-neutrophil cytoplasmic antibodies ANCA usually C-ANCA directed against Proteinase 3 (PR-3). It is postulated that auto-immune and infectious mechanisms play a role in the pathogenesis [1]. Viral, bacterial and fungal infections have been reported in association with ANCA positive vasculitis. The presence of infection suggests that pathogens may act as potential triggers of an inflammatory cascade ultimately resulting in vascular inflammation [2]. We report a case of WG that may have been triggered by blastomycosis. To our knowledge, this is the first reported case of such an association.

## Case presentation

A 16 year old previously healthy girl presented with a 3 day history of progressive pain and swelling in the right calf with inability to bear weight. On further questioning, she also complained of arthralgias, myalgias for the past month with history of intermittent chest pain in the absence of cough or dyspnea for the previous 3 months. In addition, she had generalized constitutional symptoms of generalized fatigue and malaise for the past 2 weeks. She had no history of fever, night sweats weight loss, rash, ocular, ear, nose, throat, gastrointestinal or genitourinary. These symptoms occurred 3 months following a 2 week travel to Kenora, Ontario. Physical examination was unremarkable, with the exception of a swollen warm and exquisitely tender right calf.

**Figure 1:**
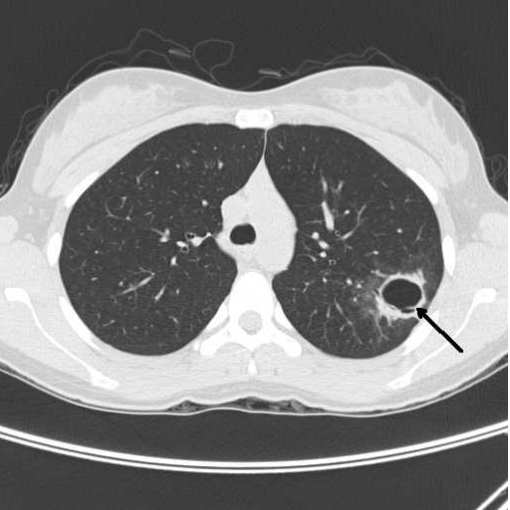
Chest CT showed a 3 cm cavitary lesion in the left upper lobe with surrounding consolidation.

A Computerized Tomography (CT) scan of the chest and an ultrasound Doppler of right leg were performed because of concern of deep vein thrombosis (DVT) and pulmonary embolism. Chest CT showed a 3 cm cavitary lesion in the left upper lobe with surrounding consolidation (figure 1). Ultrasound Doppler showed no evidence of DVT. Acute phase reactants were elevated: ESR 56 mm/hr and C reactive protein 60 mg/l. Urinalysis showed trace protein and 9 RBC/HPF with no urinary sedimentation or casts. Other initial investigations including a complete blood count, liver enzymes and creatinine kinase were normal. The initial working diagnosis was a pulmonary infection with a septic embolus in the calf musculature. The patient was treated with broad spectrum antibiotics with rapid resolution of the calf pain and swelling.

However, although she improved, she continued to have migrating polymyalgias, arthralgias and intermittent trace hematuria prompting investigations for an underlying rheumatic disease. Antinuclear antibodies including anti-double stranded DNA, and anti-extractable nuclear antigens were all negative. However, C-ANCA was positive by immunoflurescence and PR3 titer by ELSIA was strongly positive at 94 (positive levels > 30). suggestive of a diagnosis of WG. Because there was a recent travel history to Kenora, Ontario, a region with a high incidence of blastomycosis, a bronchoscopy and an open lung biopsy were preformed. The lung biopsy revealed fungal yeast forms compatible with blastomycosis, and changes of necrotizing granulomatous inflammation and vasculitis compatible with WG. The extent of the necrotizing granulomatous disease and the necrotizing vasculitis, seemed more than would be expected with blastomycosis alone.

Because of the concern that immunosuppressive therapy might lead to disseminated blastomycosis, and as there remained some question that blastomycosis alone could explain all her symptoms and laboratory findings it was elected to initially treat her with the anti-fungal agent Itraconazole alone. With this treatment there was some improvement in chest pain and the radiographic findings, but she continued to have myalgias, arthralgias, intermittent trace hematuria and persistent elevation in acute phase reactants and anti-PR3 levels. She also developed episcleritis and epistaxis. A kidney biopsy showed mildly active pauci-immune proliferative glomerulonephritis. Based on the additional system involvement the diagnosis of WG was felt to be confirmed, so after 4 months of Itraconazole, she was started on prednisone (1 mg/kg/day) and cyclophosphamide (2 mg/kg/day) with rapid improvement in her symptoms and normalization of acute phase reactants. The anti-PR3 levels also became negative.

## Discussion

Blastomycosis is a rare but potentially fatal infection by the fungus *Blastomycosis dermatitidis*. Blastomycosis is endemic in the united states around Mississippi and Ohio River basins, and Midwestern states, Canadian provinces bordering the great lakes and small areas bordering the St. Lawrence River, and Manitoba. Hyperendemicity in the region surrounding Kenora, Ontario have recently been reported with an annual incidence rate of 117.2 per 100,000 population [3]. Children account for 2–11% of cases [4,5].

The initial clinical presentation of blastomycosis includes fever, malaise, weight loss, cough and pleuritic chest pain. Cutanous lesions and less commonly bone, genitourinary and central nervous system involvement can occur. Most cases of acute blastomycosis are self-limited [6], but the mortality rate of untreated chronic blastomycosis approaches 60% [7]. The diagnosis is made by demonstration of the organism in the lesions. Most experts advocate specific targeted antifungal therapy for all cases of blastomycosis. Itraconazole is the drug of choice for patients with blastomycosis that is not life threatening, and does not involve the central nervous system. Amphotericin B is reserved for those with severe life threatening disease, central nervous system involvement and immunocompromised patients.

WG is a rare multisystem disorder, characterized by necrotizing granulomatous inflammation and pauci-immune small vessel vasculitis [8]. The triad of paranasal sinus involvement, pulmonary infiltration and renal involvement is characteristic. Up to 90% of children with WG have initial upper airway symptoms [9], such as nasal discharge, sinusitis, or epistaxis. Lower respiratory symptoms occur in 74% of children with WG [9], symptoms include cough, dyspnea, and hemoptysis. Nodular and cavitating lung lesion are often visible on radiographs. Renal disease occurs in 61% of children with WG [9] and often leads to renal failure. Disease of the skin (purpura, vesicles, papules, and nodules);CNS (cranial nerve palsy, seizures and peripheral neuropathy); heart (myocardial infarction, arrhythmia, and valvulitis); and eyes (scleritis, episcleritis, dacrocystitis, and corneal ulcers) may occur.

The diagnosis of WG can be difficult; tissue diagnosis of necrotizing granulomatous inflammation is the gold standard. The diagnosis is supported by the presence of C-ANCA (anti-PR3). Although C-ANCA has a high specificity 90–95% [10,11], it has been reported in patients with infections such as HIV, CMV Mycobacterium avium-intercellulare, invasive amebiasis, Bartonella Henessae, and Malaria. [12-18]. A positive C-ANCA can also occur in connective tissue diseases such as systemic lupus erythematosus and rheumatoid arthritis [19], as well as other vasculitides including microscopic polyangitis and Churg-Strauss syndrome [20]. Accordingly, many authorities feel that neither the presence of a positive C-ANCA nor positive PR3 antibodies should be used to replace a tissue biopsy in confirming the diagnosis of WG.

In our patient, the clinical picture of malaise, weight loss, myalgia, arthralgia, cavitating and nodular lung lesions, microscopic hematuria, episcleritis, nasal crusting and epistaxis supported by the finding of a positive C-ANCA suggested the diagnosis of WG. Open lung biopsy confirmed the diagnosis of blastomycosis and the histology was strongly suggestive of a co-existing granulomatous vasculitis. Although granulomatous changes in tissues can certainly occur with blastomycosis the extent of the vascular changes were felt to be more than could be explained by blastomycosis alone.

Although, there has been other reports of ANCA associated vasculitis triggered by fungal infection [21,22], as far as we can ascertain this is the first report of WG co-existing with tissue proven blastomycosis infection. The temporal correlation of the symptoms suggests that the fungal infection may have triggered the systemic vasculitis. The other possible explanation could be given the very high incidence of blastomycosis in Kenora region in Ontario, a patient with evolving WG could contract blastomycosis.

The unusual association of blastomycosis and WG raises the intriguing possibility that infections, particularly those in which granulomata formation occurs, may be one of the triggers for WG in the predisposed host [23]. Epidemiological evidence to support this hypothesis is presently lacking. There has been anecdotal (albeit unpublished) evidence that there was a recent increase in the occurrence of WG in children in several areas of North America, suggesting perhaps that infection can be a trigger for WG in children. The recent development of a pediatric Wegener's registry by the Childhood Arthritis and Rheumatology Research Alliance (CARRA) will hopefully allow the accrual of epidemiologic evidence that will help confirm or refute this possibility.

## Competing interests

The authors declare that they have no competing interests.

## Authors' contributions

RA, KH and PM were involved in patient care and in drafting the manuscript. SD was involved in infectious disease aspect of patient care. MS was involved in the pathology-related portion of the manuscript.

All authors read and approved the final manuscript.

## Consent

Consent for publication was obtained from the relatives (parents) of the patient.
